# The FAST-EU trial: 12-month clinical outcomes of women after intrauterine sonography-guided transcervical radiofrequency ablation of uterine fibroids

**DOI:** 10.1007/s10397-015-0915-3

**Published:** 2015-09-28

**Authors:** Hans Brölmann, Marlies Bongers, José Gerardo Garza-Leal, Janesh Gupta, Sebastiaan Veersema, Rik Quartero, David Toub

**Affiliations:** Vrije Universiteit Medisch Centrum, Amsterdam, Netherlands; Máxima Medisch Centrum, Veldhoven, Netherlands; Universidad Autónoma de Nuevo León, Monterrey, Nuevo Leon Mexico; Birmingham Women’s Hospital, Birmingham, UK; Sint Antonius Ziekenhuis, Nieuwegein, Netherlands; Medisch Spectrum Twente, Enschede, Netherlands; Gynesonics, Inc., Redwood City, CA 94063 USA; Albert Einstein Medical Center, 5501 Old York Road, Philadelphia, PA 19141 USA

**Keywords:** Fibroids, Radiofrequency ablation, VizAblate, Intrauterine sonography, Ultrasound

## Abstract

The FAST-EU Trial was designed to establish the effectiveness and confirm the safety of transcervical intrauterine sonography-guided radiofrequency ablation with the VizAblate™ System in the treatment of symptomatic uterine fibroids. This was a multicenter, prospective, single-arm trial involving academic and community hospitals in the United Kingdom, the Netherlands, and Mexico. Women with qualifying uterine fibroids and heavy menstrual bleeding underwent intrauterine sonography-guided transcervical radiofrequency ablation (RFA) with the VizAblate System; anesthesia was individualized. Patients were required to have up to five fibroids from 1 to 5 cm in diameter. The primary trial endpoint was the percentage change in perfused fibroid volume, as assessed by contrast-enhanced MRI at 3 months by an independent core laboratory. Secondary endpoints, evaluated at 6 and 12 months, included safety, percentage reductions in the Menstrual Pictogram (MP) score, and the Symptom Severity Score (SSS) subscale of the Uterine Fibroid Symptom-Quality of Life (UFS-QOL) questionnaire, along with the rate of surgical reintervention for abnormal uterine bleeding and the mean number of days to return to normal activity. Additional assessments included the Health-Related Quality of Life (HRQOL) subscale of the UFS-QOL, nonsurgical reintervention for abnormal uterine bleeding, anesthesia regimen, patient satisfaction, and pain during the recovery period. An additional MRI study was performed at 12 months on a subgroup of patients. Fifty patients (89 fibroids) underwent transcervical radiofrequency ablation with the VizAblate System. At 3 and 12 months, perfused fibroid volumes were reduced from baseline by an average of 68.1 ± 28.6 and 67.4 ± 31.9 %, respectively, while total fibroid volumes were reduced from baseline by an average of 54.7 ± 37.4 and 66.6 ± 32.1 %, respectively (all *P* < .001 compared with baseline; Wilcoxon signed-rank test). At 12 months, mean MP score and SSS decreased by 53.8 ± 50.5 and 55.1 ± 41.0 %, respectively; the mean HRQOL score increased by 277 ± 483 %. There were four surgical reinterventions (8 %) within 12 months. This is the first report of the 12-month follow-up for patients in the FAST-EU Trial. In concert with previously reported 3- and 6-month endpoint data, the 12-month results of the FAST-EU Trial suggest that in addition to substantially reducing the perfused and total volume of targeted uterine fibroids, the VizAblate System is safe and effective through 12 months in providing relief of abnormal uterine bleeding associated with submucous, intramural, and transmural fibroids.

## Introduction

Uterine fibroids are highly prevalent and the primary indication for over 200,000 hysterectomies performed annually in the USA [1, 2]. While various fibroid treatments exist, they have limitations, such as being invasive, requiring general anesthesia, or being not optimally suited for treatment of both intramural and submucous myomata.

Radiofrequency ablation (RFA) involves the placement of one or more needle electrodes into a solid tumor in order to deliver thermal energy, resulting in thermal fixation and coagulative necrosis within the treated tissue [3, 4]. Recent studies have been performed using RFA in conjunction with simultaneous, real-time sonography to guide volumetric ablations, resulting in volume reduction and symptom improvement [3, 5, 6].

The VizAblate System (Gynesonics; Redwood City, CA) combines radiofrequency ablation with intrauterine sonography and is CE-marked and commercially available in the European Union. VizAblate permits real-time imaging and transcervical treatment of uterine fibroids, including those that are not amenable to hysteroscopic resection such as type 3, type 4, and types 2–5 (transmural) fibroids as well as large type 1 and type 2 myomata [7]. The Fibroid Ablation Study-EU (FAST-EU) was designed to examine the safety and effectiveness of transcervical radiofrequency ablation of uterine fibroids under intrauterine sonography guidance with the VizAblate System. The trial endpoints, reached at 3 and at 6 months, have previously been reported [8]. This paper presents the 12-month efficacy and safety results of women treated under the FAST-EU Trial.

### Patients and methods

This was a prospective, single-arm, multicenter trial. The primary endpoint was the percentage change in target fibroid perfused volume as assessed by contrast-enhanced MRI by an independent core laboratory at baseline and at 3 months. Additional endpoints, reached at 6 months, included safety, percentage reductions in the Menstrual Pictogram (MP) score and the Symptom Severity Score (SSS) subscale of the Uterine Fibroid Symptom-Quality of Life (UFS-QOL) questionnaire, the rate of surgical reintervention for abnormal uterine bleeding, and the mean number of days to return to normal activity. The Health-Related Quality of Life (HRQOL) subscale of the UFS-QOL questionnaires, along with anesthesia regimen, patient satisfaction, and recovery pain, was also assessed.

Patients were enrolled across seven sites in three nations: Mexico (one site), the United Kingdom (two sites), and the Netherlands (four sites). The trial included women with one to five uterine fibroids of FIGO types 1, 2, 3, 4, and 2–5 (transmural) measuring between 1 and 5 cm in maximum diameter. Fibroids that did not contain an edge within the inner half of the myometrium were not counted in this total and were not targeted for ablation, as they were believed to be less likely to materially contribute to abnormal uterine bleeding (AUB). At least one fibroid was required to indent the endometrial cavity.

Patients were 28 years of age or older and not pregnant, with regular, predictable menstrual cycles and heavy menstrual bleeding for at least 3 months. A Menstrual Pictogram score ≥120 was also required for inclusion along with a baseline UFS-QOL SSS ≥20. The Menstrual Pictogram was first described by Wyatt and colleagues and is a variant of the Pictorial Blood Loss Assessment Chart (PBAC) that patients complete to provide a visual assessment of menstrual blood loss during a single cycle [9, 10]. Unlike the original PBAC described by Higham and colleagues, the Menstrual Pictogram includes a greater range of icons representing different saturations of sanitary products, clots, and losses in a toilet and also distinguishes different absorbency levels of sanitary napkins and tampons [11].

Exclusions included a desire for future fertility, the presence of one or more type 0 fibroids, cervical dysplasia, endometrial hyperplasia, active pelvic infection, clinically significant adenomyosis (>10 % of the junctional zone measuring more than 10 mm in thickness as measured by MRI), and the presence of one or more treatable fibroids that were significantly calcified (defined as <75 % fibroid enhancement by volume on contrast-enhanced MRI). Screening included transvaginal sonography, as well as hysteroscopy or hysterosonography, contrast-enhanced MRI, endometrial biopsy, and a pregnancy test.

All records were de-identified and only the range of each patient’s age was documented, as per clinical trial requirements in the Netherlands. Women were followed at 7–14 days, 30 days, 3 months, 6 months, and 12 months post-treatment. All MRI studies were forwarded to an independent core laboratory (MedQIA, Los Angeles, CA, USA) for quality control and interpretation to reduce variability in the measurements; the core laboratory also developed standardized imaging protocols for use at the individual trial sites, credentialed the sites, and trained MRI technologists at each trial site. Fibroid measurements consisted of the total voxel volume and perfused voxel volume via contrast-enhanced MRI at the specified time points.

### Procedure

**Fig. 1:**
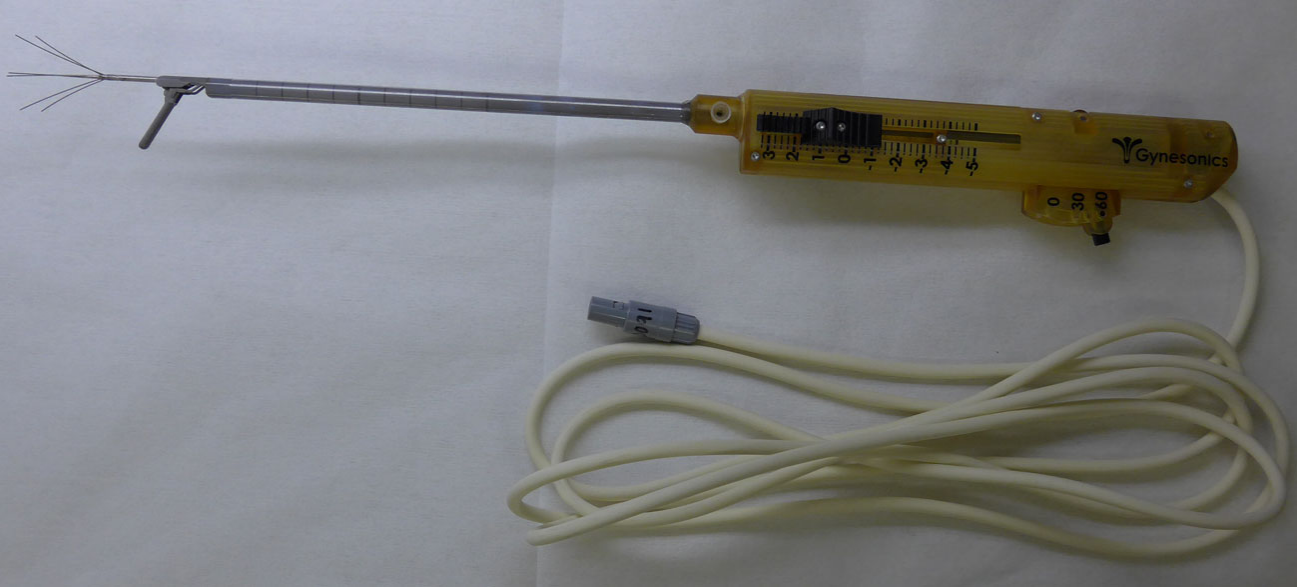
The VizAblate treatment device

The VizAblate System, as well as its use, has previously been described in detail and includes a reusable intrauterine ultrasound (IUUS) probe and a single-use, articulating radiofrequency ablation handpiece that are combined into an integrated treatment device that is inserted transcervically (Fig. 1) [8]. A custom graphical interface provides the gynecologist with a real-time, image-guided treatment system that indicates the borders of the thermal ablation (Ablation Zone) as well as the border beyond which tissues are safe from ablation (Thermal Safety Border). Because the deployment path is predictable relative to the ultrasound image, one can plan the ablation location and size before introducing any electrode elements into a fibroid. Additionally, the guidance software provides graphics that allow the gynecologist to maintain a safe margin from the ablation to the serosal margin and extrauterine viscera. Mechanical stops provide definitive tactile limits, ensuring that the needle electrodes are deployed to the proper distance to achieve the ablation size as selected by the gynecologist. The radiofrequency generator modulates power (up to 150 W) to maintain a constant temperature of 105 °C at the needle electrode tips, and the ablation time is preset based on the ablation size. Depending on the width of the ablation, the distance from the Ablation Zone to the Thermal Safety Border will vary from 6.0 to 9.5 mm.

In this trial, the method of anesthesia was chosen by each investigator based on individual patient characteristics in consultation with an anesthesiologist. Treated fibroids received one or more ellipsoidal ablations under real-time intrauterine sonographic guidance, ranging from 1 to 4 cm in width and 2 to 5 cm in length. The number of ablations, along with their sizes, was at the discretion of the investigator and was chosen in order to maximize the ablation volume of the fibroid while maintaining the Thermal Safety Border within the uterine serosal margin.

### Statistical analysis

The primary endpoint was the percentage change in target fibroid perfused volume at 3 months. The null hypothesis for the primary trial endpoint at 3 months was H_0_: probability of success <50 % versus the alternative H_a_: probability of success ≥50 %. A sample of 40 patients was sufficient to detect this difference of 22 % in probability of success with a power of 82 % using a one-group chi-square test with a 0.05 two-sided significance level. Allowing for an expected dropout rate of 20 % at the 12-month follow-up visit, the minimum recommended sample size for the initial trial protocol was 48. The primary trial endpoint success criterion was achievement of >30 % reduction in mean target fibroid perfused volume in at least 50 % of patients at 3 months.

The data in this report consist of the Full Analysis dataset. This includes all patients enrolled who provided a baseline fibroid volume assessment and received treatment with the VizAblate System. Patients who received a surgical reintervention were considered treatment failures, and their subsequent data was imputed using the last observation carried forward (LOCF) method. Missing data was not imputed for patients who conceived or who neglected to complete a questionnaire.

All statistical analyses were performed with SAS 9.3 (SAS, Cary, NC). Values were considered significant at the level of α = 0.05. The Wilcoxon signed-rank test was used to test if a change was significantly different from 0.

### Ethics

The protocol was approved by the Ethics Committees of the respective institutions as well as by the Federal Commission for Protection against Health Risks (COFEPRIS) in Mexico. All enrolled patients provided written informed consent for treatment with the VizAblate System prior to enrollment. The trial overview was published on ClinicalTrials.gov (identifier: NCT01226290) and conducted in accordance with Standard ISO 14155 (Clinical investigation of medical devices for human subjects – Good clinical practice) of the International Organization for Standardization (ISO), the Helsinki Declaration of 1975, as revised in 2008, and the ethical standards of applicable national regulations and institutional research policies and procedures governing human experimentation.

## Results

### Patients

Fifty patients were treated in the FAST-EU trial at seven sites. Baseline characteristics for all treated patients are provided in Table 1. Anesthesia was provided as noted in Table 2.

**Table 1:** Baseline subject characteristics

Subjects treated	50
Most frequent age range	41–45 years of age^a^
Mean Menstrual Pictogram (MP) score	423 ± 253 (range 119–1582)
Mean UFS-QOL SSS	61.7 ± 16.9 (range 28.1–100.0)
Mean UFS-QOL HRQOL score	34.3 ± 19.0 (range 0.0–73.3)
Total number of target fibroids identified on MRI	118
Mean number of target fibroids per patient	2.4 ± 1.7 (range 1–7)^b^
Mean diameter of target fibroids	2.9 ± 1.4 cm (range 1.0–6.9 cm)
Mean perfused fibroid volume	18.3 ± 20.6 cm^3^ (range 0.3–77.0 cm^3^)
Mean total (perfused + nonperfused) fibroid volume	18.8 ± 21.4 cm^3^ (range 0.3–77.0 cm^3^)

*UFS-QOL* Uterine Fibroid Symptom-Quality of Life Questionnaire, *SSS* Symptom Severity Score subscale, *HRQOL* Health-Related Quality of Life subscale

^a^Subject ages were specified as a range by each site to protect subject privacy

^b^Two small additional fibroids, beyond the upper limit of 5 target fibroids/patient, were identified on review of one MRI series after treatment

**Table 2:** Anesthesia provided to FAST-EU subjects

Anesthesia option	No. of subjects
General anesthesia alone	15 (30.0 %)
Conscious sedation alone	15 (30.0 %)
Spinal anesthesia alone	8 (16.0 %)
Conscious sedation + epidural anesthesia	8 (16.0 %)
Epidural anesthesia alone	2 (4.0 %)
Paracervical blockade alone	1 (2.0 %)
General anesthesia + epidural anesthesia	1 (2.0 %)

**Fig. 2:**
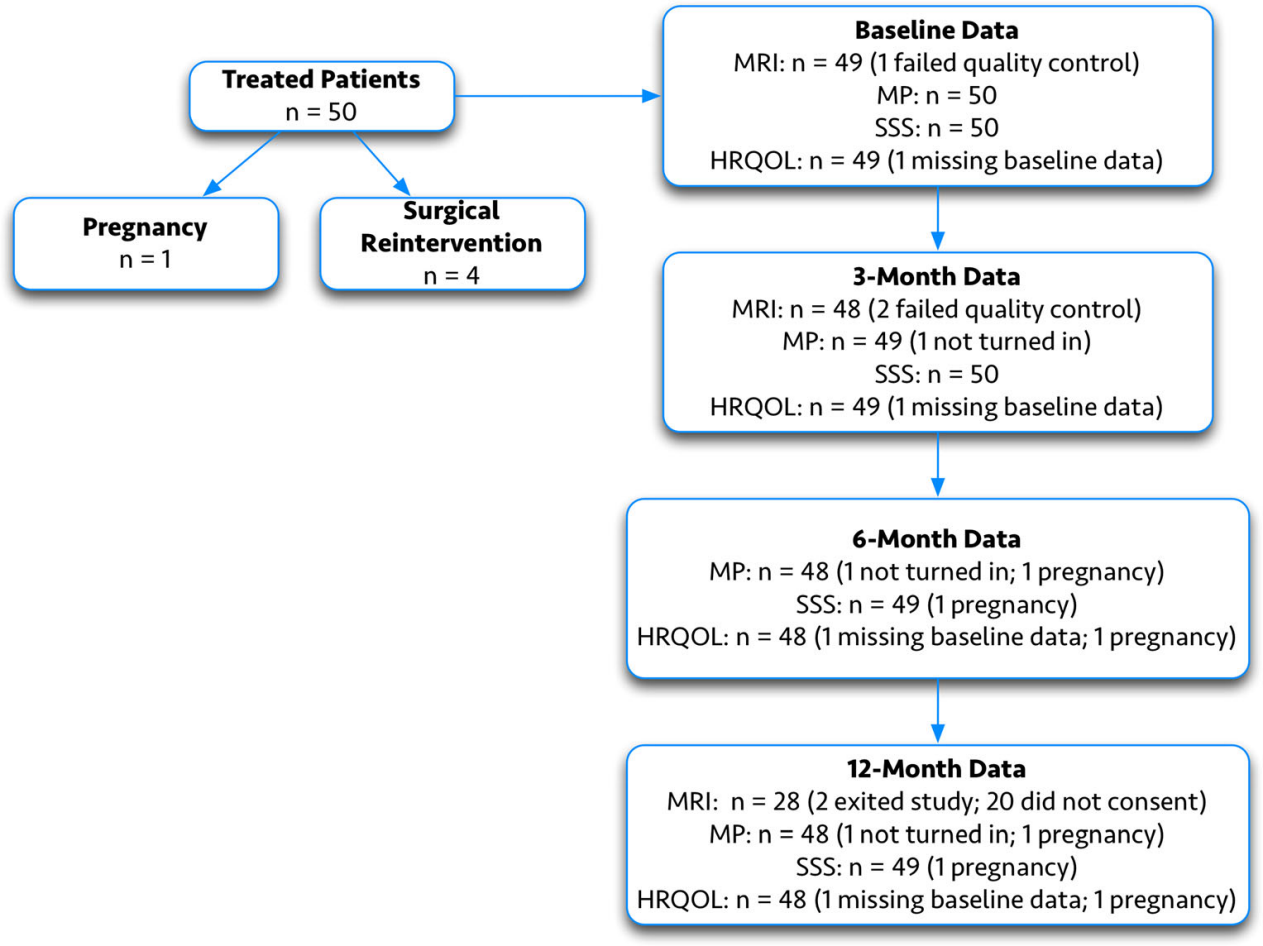
Patient flow diagram

One patient (three fibroids) was excluded from analysis of the primary endpoint. This patient was deemed by the core MRI laboratory to have had unusable imaging for making precise baseline fibroid measurements, although eligibility based on fibroid diameter ≤5 cm and location was not in question. This patient was treated as she met the eligibility requirements and her treatment could contribute to patient-reported and safety data for the trial. Consequently, while 92 fibroids were ablated, accurate baseline volume measurements could only be performed for 89. One patient reported a pregnancy at the time of her 6-month follow-up visit and was thus excluded from the 6- and 12-month analyses. While all patients provided baseline MP data, one patient each at 3, 6, and 12 months declined to submit a Menstrual Pictogram. One patient did not turn in her baseline HRQOL portion of the UFS-QOL; her HRQOL data was not included in the analysis. A flow diagram depicting sample sizes for MRI and patient-reported outcomes at baseline and 3-, 6-, and 12 months is provided in Fig. 2.

The protocol required a baseline and 3-month MR study for the primary endpoint analysis (reduction in perfused fibroid volume). Approximately 14 months after the first patient was treated, the protocol was amended to add an MR evaluation at 12 months in order to provide longer-term information about the effects of transcervical RFA. Twenty-eight patients (58.3 %) provided their informed consent to undergo another MR examination with contrast enhancement at 12 months post-ablation and underwent such imaging.

### Effects on fibroid volume

Characteristics of fibroids that were ablated are shown in Table 3, and results of fibroid ablation on total and perfused volume at 3 and 12 months are provided in Table 4. Fibroids are classified in Table 3 as per the FIGO classification system [12]. Radiofrequency ablation with the VizAblate System was associated with statistically significant reductions (68.1 and 54.7 %, respectively) in both total and perfused fibroid volumes at 3 and 12 months. Seventy-nine of 89 treated fibroids (88.8 %) in all 49 patients with measurable MRI data met the primary trial endpoint success criterion at 3 months (achievement of >30 % reduction in mean target fibroid perfused volume at 3 months in at least 50 % of patients). By 12 months post-ablation (*n* = 28 patients; 43 fibroids), treated fibroids experienced a mean reduction in total fibroid volume of 66.6 ± 32.1 % (*P* < .001). Thirty-seven fibroids (86.0 %) in 100 % of the 28 patients imaged at 12 months demonstrated >30 % reduction in perfused fibroid volume at 12 months.

**Table 3:** Characteristics of ablated fibroids

Total number of ablated target fibroids^a^	92
Mean number of ablated target fibroids per subject	1.8 ± 1.1 (range 1–5)
Total number of type 0 ablated fibroids	0
Total number of type 1 ablated fibroids	14
Total number of type 2 ablated fibroids	42
Total number of type 3 ablated fibroids	3
Total number of type 4 ablated fibroids	25
Total number of type 2–5 (transmural) ablated fibroids	8
Mean diameter of ablated fibroids	3.2 ± 1.4 cm (range 1.1–6.9 cm)

^a^Includes three fibroids that were ablated in a subject whose MRI data was not evaluable with regard to precise fibroid measurements

**Table 4:** Reduction in mean perfused and total fibroid volumes through 12 months

	Baseline	3 months	% Reduction from baseline	*P* value^a^	12 months^b^	% Reduction from baseline	*P* value^a^
No. of ablated fibroids	89	89			43		
No. of subjects	49	49			28		
Perfused fibroid volume (cm^3^)	18.3 ± 20.6 9.5 (0.3–77.0)	5.8 ± 9.6 1.6 (0.0–45.7)	68.1 ± 28.6 % 76.9 % (−33.3 to 100 %)	<.001	6.6 ± 11.3 1.0 (0.0–56.1)	67.4 ± 31.9 % 73.3 % (−32.7 to 100 %)	<.001
Total fibroid volume (cm^3^)	18.8 ± 21.4 9.5 (0.3–77.0)	8.0 ± 12.0 1.9 (0.0–56.3)	54.7 ± 37.4 % 62.5 % (−85.7 to 100 %)	<.001	6.8 ± 11.4 1.2 (0.0–56.1)	66.6 ± 32.1 % 73.3 % (-32.7–100 %)	<.001

Data are mean ± standard deviation; median (range)

^a^Wilcoxon signed-rank test, null hypothesis of no change

^b^A 12-month MRI study was added through a protocol amendment after several patients had been treated, and 28 patients provided informed consent to undergo this additional imaging study

### Patient-reported outcomes

Patient-reported secondary endpoint data through 12 months are provided in Table 5. The mean MP score declined through 12 months, with mean and median reductions of 53.8 and 72.3 % at 12 months, respectively (all *P* < .001). By 3 months post-ablation, 44 of 49 patients (89.8 %) experienced a reduction in menstrual blood loss as reflected by their Menstrual Pictogram scores. Of these 49 patients at 3 months, 28 (57.1 %) had >50 % reduction in MP scores; this proportion increased to 35 of 48 patients (72.9 %) at 6 months and was realized by 31 of 48 patients (64.6 %) at 12 months. The proportion of patients achieving >50 % bleeding reduction at 6 months was not significantly different from the proportion at 12 months (*P* = .095).

**Table 5:** Improvement in patient-reported outcomes through 12months

	Baseline	3months	Change from baseline	% Change from baseline	*P* value^a^	6months	Change from baseline	% Change from baseline	*P* value^a^	12 months	Change from baseline	% Change from baseline	*P* value*
MP	50 423 ± 253 361 (119–1582)	49 202 ± 202 170 (0–1011)	49 221 ± 290 191 (−700, 1265)	49 45.2 ± 57.9% 56.9% (−225–100%)	<.001	48 181 ± 209 107 (0–1011)	48 244 ± 302 191 (−700, 1307)	48 51.9 ± 59.8% 68.6% (−225–100%)	<.001	48 173 ± 200 85 (0–786)	48 243 ± 296 217 (−343, 1543)	48 53.8 ± 50.5% 72.3% (−103–100%)	<.001
SSS	50 61.7 ± 16.9 60.9% (28.1–100%)	50 31.7 ± 20.1 31.3% (0.0–93.8%)	50 30.0 ± 22.2 31.3 (−18.8, 84.4)	50 46.7% ± 32.8% 52.5% (−33.3–100%)	<.001	49 25.1 ± 19.3 18.8 (0.0–78.1)	49 36.7 ± 22.6 37.5 (−6.3, 75.0)	49 57.6 ± 31.4% 66.7% (−22.2–100%)	<.001	49 26.6 ± 24.0 21.9 (0.0–78.1)	49 35.3 ± 26.9 37.5 (−18.8, 93.8)	49 55.1 ± 41.0% 62.5% (−66.7–100%)	<.001
HRQOL	49 34.3 ± 19.0 30.2 (0.0–73.3)	49 76.4 ± 22.2 83.6 (5.2–100)	49 42.1 ± 25.6 40.5 (−7.8, 95.7)	49 336 ± 846% 123% (−11.1–5550%)	<.001	48 79.5 ± 22.7 85.3 (0.9–100)	48 44.5 ± 26.7 45.3 (−5.2, 96.6)	48 266 ± 475% 118% (−28.6–2800%)	<.001	48 80.7 ± 24.7 91.4 (0.9–100)	48 45.7 ± 30.5 45.7 (−33.6, 96.6)	48 277 ± 483% 127% (−54.2–2800%)	<.001

Data are number of subjects; mean ± standard deviation; median (range)

*MP* Menstrual Pictogram, *SSS* Symptom Severity Score, *HRQOL* Health-Related Quality of Life

^a^Wilcoxon Signed-Rank Test, null hypothesis of no change

Lukes and colleagues reported that a 22 % or greater reduction in menstrual blood loss was meaningful to the majority of women [13]. In the FAST-EU Trial, 37 of 49 (75.5 %) patients had achieved such clinically meaningful reductions in menstrual bleeding by 3 months. This increased to 41 of 48 patients (85.4 %) at 6 months and 38 of 48 patients (79.2 %) at month 12, which was not significantly different from 6 months (*P* = .175).

As shown in Table 5, the reductions in the transformed SSS subscale of the UFS-QOL questionnaire at 3, 6, and 12 months were statistically significant, as were the increases in the transformed HRQOL subscale. Patients experienced a 55.1 % reduction in SSS at 12 months, corresponding to a mean reduction in transformed SSS of 35.3 points from baseline. At all post-ablation time points studied, the majority of patients experienced at least a clinically significant 10-point reduction in SSS (82 % of patients at 3 months, 86 % at 6 months, 78 % at 12 months).

### Adverse events

There were 34 adverse events deemed possibly, probably, or definitely related to the VizAblate System or overall procedure over a 12-month period. These included seven women with dysmenorrhea, six with abnormal uterine bleeding above baseline, four with pelvic pain and/or cramping, two urinary tract infections (both within 30 days of treatment), and one fibroid expulsion that had no significant consequences. There were two readmissions within 30 days of the procedure. One patient was admitted overnight on post-procedure day #9 to receive parenteral antibiotics for lower abdominal pain believed secondary to cystitis (one of the two instances of urinary tract infection previously noted) and was discharged on the following day. Another patient developed bradycardia down to 38 bpm shortly after the procedure and was kept overnight in the hospital for successful treatment with atropine and observation.

### Surgical reintervention

Four patients (8 %) underwent surgical reintervention, all after 6 months post-ablation. One patient underwent hysteroscopy and nonresectoscopic endometrial ablation (ThermaChoice®; Ethicon, Somerville, NJ) at 10 months. At the time of her endometrial ablation, hysteroscopy confirmed the presence of a normal endometrial cavity; no residual fibroid tissue was noted. Two patients, both treated by the same investigator, underwent hysteroscopic myomectomy at 6.5 and 7 months post-ablation, respectively, due to AUB felt secondary to fibroid sloughing. In both cases, the ablated fibroids had a 70–85 % reduction in perfused volume at 3 months. A fourth patient underwent total abdominal hysterectomy at 11 months secondary to abnormal uterine bleeding above baseline. The patient was noted post-operatively to have had an abnormal bleeding duration at baseline that had not been reported in her menstrual history, constituting a protocol violation. The patient may have had a component of anovulation contributing to her abnormal uterine bleeding.

### Pregnancy

There was a single pregnancy reported within the first 6 months after ablation with the VizAblate System. The patient presented with 12 weeks of amenorrhea at her 6-month trial visit, had a positive pregnancy test at that time, and delivered a live-born male infant at term via elective repeat Cesarean section [14].

### Return to normal activity, patient satisfaction, and pain during recovery

Forty-eight patients provided results of a 10-point visual analogue scale (VAS) regarding their pain during the recovery period (up to 14 days post-treatment). On average, they reported a mean VAS score of 3.0 ± 1.7 (median 3.0, range 0–9). Forty-seven patients completed a recovery diary relating to how long it took them to return to their normal activities of daily life. On average, return to normal activity took 4.4 ± 3.1 days (median 4.0 days, range 1–14 days). There was an overall satisfaction rate of 87.8 % (43/49 patients) at 12 months; 69.4 % were “very satisfied,” 10.2 % were “satisfied,” and 8.2 % were “somewhat satisfied,” with their treatment. At 12 months, 49 patients provided a mean scoring of 8.8 ± 2.4 out of 10 in terms of how likely they would be to recommend the treatment to a friend or relative.

## Discussion

It is of particular importance to determine how well patients fared beyond the previously reported 3- and 6-month endpoints from the FAST-EU Trial. The results outlined in this report confirm and extend the results of the 3- and 6-month endpoints and demonstrate that intrauterine sonography-guided transcervical radiofrequency ablation of fibroids provides significant reductions in fibroid volume and bleeding symptoms through 12 months.

Transcervical radiofrequency ablation avoids many of the potential complications associated with a laparoscopic or open procedure for the treatment of fibroids. There are no incisions, eliminating the potential for wound infection, seroma, and hematoma. The peritoneal cavity is not entered nor is the serosa penetrated or coagulated, so that intraperitoneal adhesiogenesis is unlikely. There is no overt risk of ureteral injury, unlike hysterectomy. In contrast to operative hysteroscopy, only a small quantity of hypotonic fluid is used for acoustic coupling, no large venous sinuses are exposed, and intrauterine pressure is not raised to levels above mean arterial pressure, avoiding the risk of significant fluid intravasation. The integral intrauterine sonography probe permits real-time visualization of the myometrium and serosa, providing a perspective of the myometrium and intramyometrial pathology that are not achievable with a hysteroscope and enabling treatment of intramural and transmural fibroids as well as larger submucous myomata.

The trial success criterion was >30 % reduction in mean target fibroid perfused volume at 3 months in at least 50 % of patients. This success criterion stems from the MR-guided focused ultrasound data of Stewart and colleagues, which found that sustained relief of fibroid symptoms up to 24 months is associated with nonperfused volume ratios >20 % after hyperthermic ablation [15]. Initially, it was not known if total fibroid volume would be significantly reduced at that early time point, which was the rationale for using reduction in perfused fibroid volume (measured via contrast-enhanced MRI) as the primary endpoint as opposed to reduction in total fibroid volume. In the FAST-EU Trial, contrast-enhanced MRI demonstrated significant mean reductions at 3 months in both the volume of perfused fibroid tissue as well as in total fibroid volume at 3 months (68.1 and 54.7 %, respectively). At 12 months, patients demonstrated significant reductions (67.4 and 66.6 %, respectively) in mean perfused and total fibroid volumes. It has been previously demonstrated that hyperthermic ablation of >20 % of a fibroid may provide sustained relief from fibroid symptoms [15].

There were statistically significant reductions in menstrual blood loss, as evidenced by 45.2, 51.9, and 53.8 % reductions in the menstrual pictogram at 3, 6, and 12 months, respectfully, as well as significant improvements in both subscales of the UFS-QOL questionnaire. The majority of patients (57.1–72.9 %, depending on time point) realized more than a 50 % reduction in their menstrual pictogram scores, with 75.5 % of patients achieving a clinically meaningful reduction in menstrual bleeding as early as 3 months after treatment. Similarly, 78–86 % patients realized at least a 10-point reduction in SSS (depending on time point), with a mean reduction from baseline of 35.3 points at 12 months; a 10-point reduction in SSS represents a moderate effect size and was required by the US Food and Drug Administration for the approval of MRgFUS [16].

Patients typically experienced mild or no pain through the first post-ablation visit. Return to normal activity was just over 4 days and patient satisfaction was high (87.8 %). Two patients (4 %) were hospitalized overnight, one for abdominal pain secondary to apparent cystitis and the other for observation after bradycardia that responded to atropine. Neither event was deemed to have been related to the VizAblate System upon review by an independent medical advisory board.

This trial has several noteworthy attributes. Care was taken to exclude women with abnormal uterine bleeding secondary to anovulation through strict adherence to the inclusion criterion regarding the menstrual history. Additionally, at least one fibroid was required to have indented the endometrial cavity, making it more likely that a patient’s bleeding symptoms are largely or exclusively secondary to fibroids rather than another etiology. A core MRI facility was used to reduce variability and bias in MRI imaging quality, interpretation, and measurements relative to the primary trial endpoint. In addition, the use of multiple clinical sites included academic medical centers as well as community hospitals to provide a more realistic assessment of the use of the VizAblate System in different treatment locations.

As a nonrandomized single-arm trial that does not directly compare against another fibroid treatment, this trial cannot be used to compare treatment with VizAblate to standard fibroid therapy. Only a subset of patients (28/48 eligible; 58.3 %) underwent MRI at 12 months. Finally, follow-up was limited to 12 months; longer surveillance and greater numbers of patients will be required to establish definitive efficacy and safety data. Toward that end, a larger clinical trial is underway.

## Conclusions

These results from the FAST-EU Trial demonstrate that the initial endpoint results reported at 3 and 6 months were sustained in the treated population through 12 months. Patients realized significant reductions in perfused and total fibroid volume, menstrual bleeding, overall symptoms, and improvements in quality of life. The data demonstrate the potential of intrauterine sonography-guided, transcervical radiofrequency ablation with the VizAblate System as a promising uterus-preserving technology for the treatment of submucous, intramural, and transmural fibroids without incisions or the need for general anesthesia.
